# Determination of *HER2* Amplification Status on Tumour DNA by Digital PCR

**DOI:** 10.1371/journal.pone.0083409

**Published:** 2013-12-26

**Authors:** Isaac Garcia-Murillas, Maryou Lambros, Nicholas C. Turner

**Affiliations:** 1 The Breakthrough Breast Cancer Research Centre, Institute of Cancer Research, London, United Kingdom; 2 Breast Unit, Royal Marsden Hospital, London, United Kingdom; Naval Research Laboratory, United States of America

## Abstract

Determination of the presence of *HER2* amplification by quantitative PCR has been challenging, in part due to chromosomal instability and identification of a robust a reference region. We assessed the potential of digital PCR for highly accurate assessment of DNA concentration with *EFTUD2* as chromosome 17 reference probe. We assessed a *HER2*:*EFTDU2* ratio by digital PCR assay in the microdissected DNA from 18 *HER2* amplified and 58 *HER2* non-amplified cancers. The *HER2:EFTUD2* ratio had high concordance with conventionally defined *HER2* status with a sensitivity of 100% (18/18) and a specificity of 98% (57/58). The *HER2:EFTUD2* digital PCR assay has potential to accurately assess *HER2* amplification status.

## Introduction

Treatments directed at *HER*2 have transformed the outcome of *HER2* amplified cancers [1]. Determination of the presence of *HER2* amplification in clinical practice uses both immunohistochemistry, such as the Hercept® test, to detect *HER*2 over-expression or *in situ* hybridization to assess *HER2* gene copy number [2]. The somatic genetic events that drive breast cancer have now been well described [3], with multiple clinical trials underway directed against somatic genetic events such as mutation of *PIK3CA.* Testing for such mutations is likely to become part of routine practice, which will require routine extraction of DNA, and this emphasizes the potential utility of robust DNA based assays of *HER2* amplification status, through accurate quantification of *HER2* gene copy number in extracted DNA.

Multiple prior studies have assessed *HER2* copy number in extracted DNA, although high accuracy sufficient for clinical use has been challenging to achieve [4], [5]. In part this reflects inherent limitations in the accuracy of traditional real-time PCR that can be improved through the use of digital PCR [6], [7]. However, at least in part, the difficulties achieving a highly accurate test reflect chromosomal instability in breast cancer, and the difficulty in identifying a single region in the genome to act as a robust reference region. Analysis of *HER2* mRNA over-expression has been reported to have high diagnostic accuracy [4], [8], although attempts to bring such *HER2* RNA assessments to routine practice have met mixed results; analysis of *HER2* status from the Oncotype DX^®^ 21 gene recurrence score has been reported to have high diagnostic accuracy in some series [9], with discordance in other series [10].

Here we bring together a number of recent advances to deliver a highly robust assay for *HER2* status on tumour DNA. We have previously identified a highly robust reference region for *HER2* copy number assessment, and developed a digital PCR assay to accurately assess *HER2* amplification status from extracted DNA. Here we show that this assay has very high accuracy in defining *HER2* amplification status from tumour DNA samples.

## Materials and Methods

### Patient Cohort

Tumour samples were from two previously published series of breast cancers [11], [12], [13]. Tumour samples were from fresh frozen material, microdissected to achieve at least 70% tumour cell content under a stereomicroscope prior to DNA extraction. DNA was extracted using Qiagen DNeasy Blood and Tissue Kit as per manufacturer’s instruction, and quality and quantity was assessed using Life Technologies Quant-iT™ PicoGreen® dsDNA Assay Kit as per manufacturer instructions. Clinicopathological details of the samples included in this study are listed in Table 1. *HER2* status was defined according to ASCO-CAP guidelines, and was blinded to analysis of samples by digital PCR.

**Table 1:** Clinicopathalogical details of tumours included in the study.

	All Patients
n	76
median age	61.08 (33-89)
ER positive	55
PR positive	50
HER2 positive	18
ck 5/6 positive	10
*Stage*	
I	31
II	25
III	5
IV	13
N/A	2
*Grading*	
I	5
II	27
III	44
*Pathology*	
Ductal	53
Lobular	14
Other	9

### Identification of Reference Region on Chromosome 17

We utilised microarray comparative genomic hybridisation data from 311 invasive breast cancers, 65 *HER2* amplified and *246 HER2* non-amplified [14], to identify an optimal chromosome 17 copy number reference region [15]. The copy number ratio between the mean of probes covering *HER2* and every possible reference probe on chromosome 17 was assessed for each cancer. For each possible reference probe the sensitivity for comparing amplified and non-amplified cancers was calculated, as was the statistical difference between *HER2* amplified and non-amplified cancers with the Student’s T test. The sensitivity was modeled as the proportion of *HER2* amplified cancers that had a copy number ratio higher than the maximum ratio of the *HER2* non-amplified cancers. All genomic positions were according to genome version hg19.

### Digital PCR

Digital PCR was performed as previously described [15] on a QX100 droplet digital PCR system (Bio-Rad) with *HER2* primers (HER2F: ACAACCAAGTGAGGCAGGTC, HER2R: GTATTGTTCAGCGGGTCTCC, HER2 MGB probe: FAM-CCCAGCTCTTTGAGGACAAC) at a final concentration of 900 nM primers and 250 nM probe, *EFTUD2* primers (*EFTUD2*F: GGTCTTGCCAGACACCAAAG, *EFTUD2*R: TGAGAGGACACACGCAAAAC, *EFTUD2* MBG probe: VIC-GGACATCCTTTGGCTTTTGA) at a final concentration of 900 nM primers and 250 nM probe. Primers and probes were designed bioinformatically using Primer3 (http://frodo.wi.mit.edu/). Individual primer sets were assayed by PCR and gel-electrophoresis to test for primer-dimers and non-specific product amplification. The melting temperature for digital PCR was optimized by gradient both in singleplex and multiplex. The rate of droplets positive for both *HER2* and *EFTUD2* did not exceed that expected by chance alone, assessed from the Poisson distribution (data not shown), confirming that digestion or fragmentation of DNA was not required prior to digital PCR.

PCR reactions were prepared with 5–20 ng DNA and Bio-Rad 2x ddPCR supermix for probes (Cat number 1863010) in a total volume of 20 µl, and partitioned into ∼14,000 droplets per sample in a QX100 droplet generator according to manufacturer’s instructions. DNA was diluted, when required, on nuclease free water. Emulsified PCR reactions were run on a 96 well plate on a G-Storm GS4 thermal cycler incubating the plates at 95°C for 10 min followed by 40 cycles of 95°C for 15 sec and 60°C for 60 sec, followed by 10 min incubation at 98°C. The temperature ramp increment was 2.5°C/sec for all steps. Plates were read on a Bio-Rad QX100 droplet reader using QuantaSoft v1.3.2.0 software from Bio-Rad to assess the number of droplets positive for *HER2*, *EFTUD2*, both or neither. At least two negative control wells with no DNA were included in every run.

### Digital PCR Analysis

The concentration of *HER2* DNA (copies of *HER2* DNA per droplet) was estimated from the Poisson distribution. Number of *HER2* copies per droplet M*_HER2_* = −ln (1−(n*_HER2_*/n)), where n*_HER2_* = number of droplets positive for *HER2*-FAM probe and n = total number of droplets. Similarly, number of reference probe copies per droplet M*_EFTUD2_* = −ln (1−(n*_EFTUD2_*/n)), where n*_EFTUD2_* = number of droplets positive for *EFTUD2*-VIC probe. The *HER2:EFTUD2* copy number ratio = M*_HER2_*_/_M*_EFTUD2_*. The confidence intervals for the *HER2:EFTUD2* ratio were calculated from the above equation using methods previously described [16]. We aimed for at least 400 droplets positive for *EFTUD2* to accurately assess the ratio, as at this DNA concentration a sample with a *HER2:EFTUD2* ratio of 2.2 would have a lower 95% confidence interval of 2.0 [2].

### Statistical Analysis

All other statistical analysis was two sided and performed with GraphPad Prism version 5.0 or Microsoft Excel.

## Results

**Figure 1:**
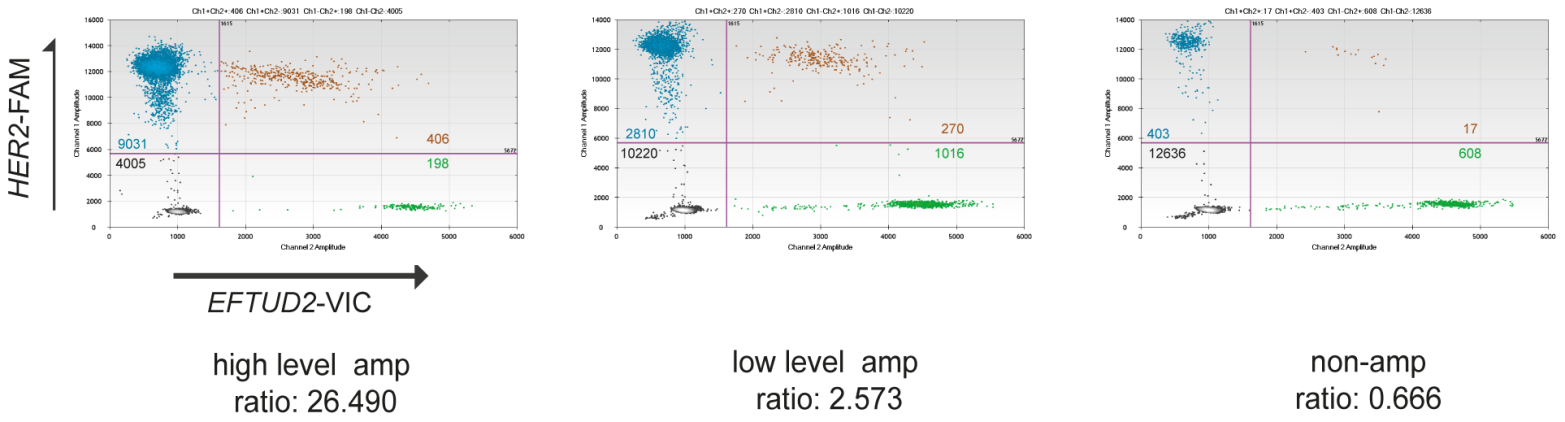
*HER2:EFTUD2* digital PCR for determinant of *HER2* status. Representative droplet digital plots from a tumour with high level amplification (left panel), low level amplification (middle panel) and a non-amplified tumour (right panel). The four quadrants represent top left: droplets with *HER2* DNA only, top right: droplets with both *HER2* and *EFTUD2* DNA, bottom right: droplets with *EFTUD2* DNA only, and bottom left: droplets with no DNA.

We previously described the bioinformatic development of a digital PCR assay for *HER2* copy number. In order to accurately report *HER2* status without false positive results due to loss of the control region or gain of the 17q chromosomal arm, we identified an optimal control region on chromosome 17 [15]. We identified *EFTUD2* on chromosome 17q21.31 as a robust copy number comparator (Figure S1). This region was very rarely co-amplified with *HER2* in amplified cancers, yet in non-amplified cancers robustly had the same copy number as *HER2*. We optimized *HER2* and *EFTUD2* primer-probes with TaqMan chemistry labeled with FAM and VIC respectively, and optimized conditions for droplet digital PCR (Figure 1).

We assessed the potential of the *HER2* digital PCR assay to differentiate *HER2* amplified and non-amplified breast cancers. We firstly assayed 11 samples in replicate during two different experiments to check for the ability of our assay to differentiate *HER2* status and also to check for reproducibility Table S1). We then assessed a series of 76 primary breast cancers described in Table 1. DNA was extracted from fresh frozen material following microdissection under a stereomicroscope to achieve >70% tumour cell content. Digital PCR was performed for each sample blinded to *HER2* status. The median *HER2:EFTUD2* copy number ratio in *HER2* amplified cancers (7.0, range 2.04–26.5) was significantly higher than in *HER2* non-amplified (1.07, range 0.53–2.00, p<0.0001 Mann Whitney U test), with the receiver operator curve area under the curve of 1.0 (95% CI undefinable).

We analysed the data with a threshold for the *HER2:EFTUD2* ratio of 2.0 to define *HER2* amplification consistent with ASCO-CAP guidelines for *HER2:CEP17* ratio [2]. The *HER2* digital PCR assay had 100% Sensitivity (18/18) and 98% Specificity (57/58). The accuracy of 99% reflected a single *HER2* non-amplified cancer by FISH that was assigned as HER2 positive by digital PCR.

## Discussion

**Figure 2:**
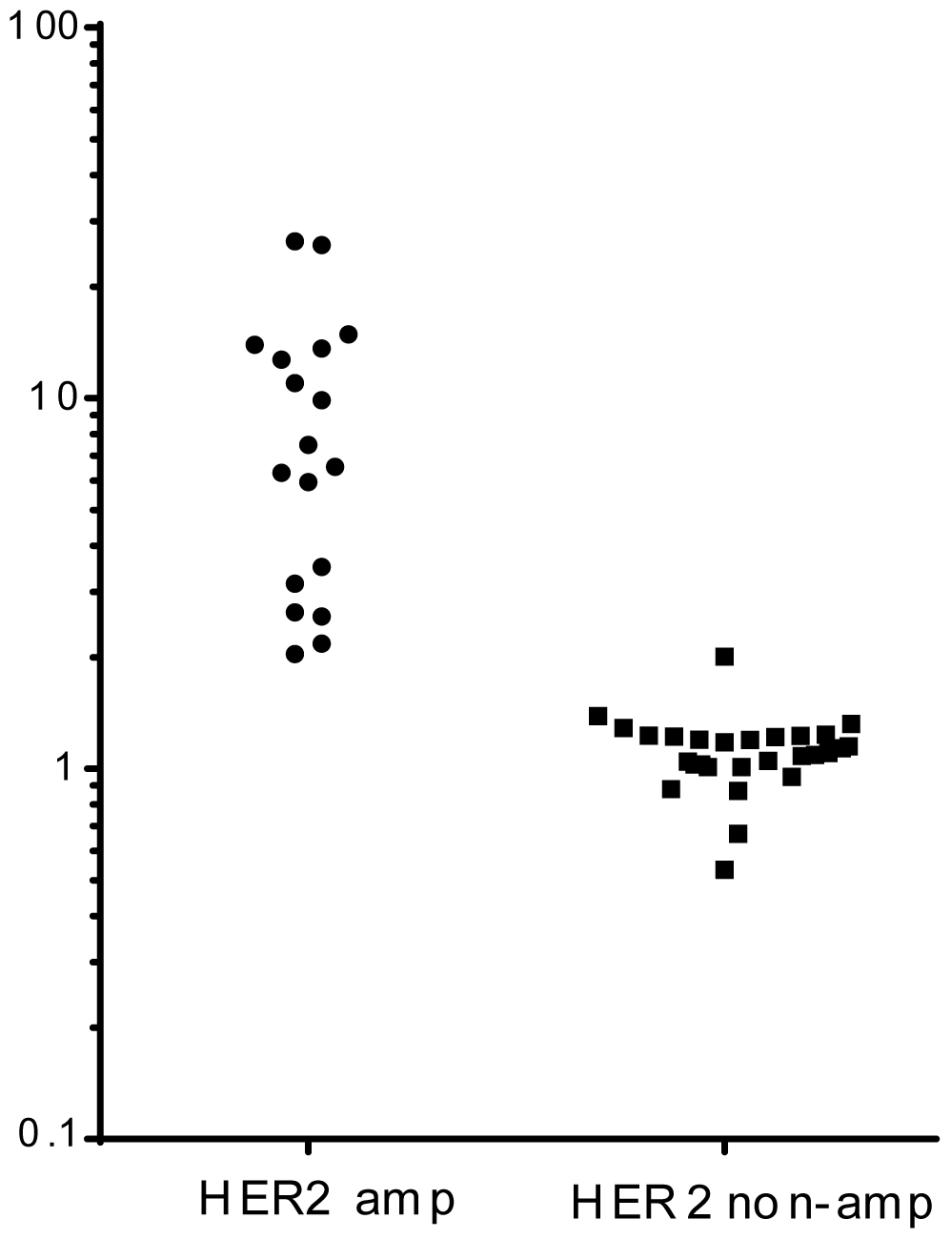
*HER2:EFTUD2* digital PCR has high accuracy compared to conventionally defined *HER2* status. *HER2:EFTUD2* ratio was assessed by digital PCR on DNA from 18 *HER2* amplified and 58 *HER2* non-amplified cancers demonstrating the narrow range of *HER2:EFTUD2* ratios in non-amplified cancers.

We demonstrate that digital PCR with *HER2:EFTUD2* ratio assessed on microdissected tumour DNA has high concordance with conventionally defined *HER2* status (Figure 2), and presents a potential option to define *HER2* status. The accuracy of the approach exploits both the accuracy of digital PCR for quantification of DNA concentration and the identification of a robust control region for copy number assessment [15].

We identified *EFTUD2* as an optimal control region for *HER2* copy number assessment. *EFTUD2*, being approximately 5 Mb telomeric to *HER2*, is sufficiently close to *HER2* in non-amplified cancers to have robustly the same copy number as *HER2*. Therefore, the specificity is not compromised by chromosomal instability, which potentially complicates assessment based on reference probes on chromosomes other than 17, or on more distal probes such as peri-centromeric probes. The extent and size of amplicons are not entirely random, driven both by co-amplification of genes that contribute to the oncogenicity of the amplicon and the less studied effects of genome structure on the extent of the amplicon boundaries. The *HER2* amplicon does not extend to the *EFTUD2* locus, and this therefore maintains the sensitivity of the assay (Figure S1 and Figure S2). Further enhancing the accuracy of the approach, the *EFTUD2* locus is frequently subject to heterozygous loss in *HER2* amplified cancers (Figure 1), which therefore enhances the *HER2*:*EFTUD2* ratio in amplified cancers. As such it must be emphasised that the *HER2*:*EFTUD2* ratio does not necessarily reflect an assessment of absolute copy number of the *HER2* locus, but is a potential diagnostic test for the presence of the amplification.

The DNA samples assessed in this study were microdissected to achieve >70% tumour DNA content. Our results suggest that the digital PCR assay has the potential to be used with less strict microdissection, and this could be assessed in future studies. The *HER2:EFTUD2* ratio range was narrow in non-amplified cancers, with only one of 58 cancers having a *HER2:EFTDU2* ratio >1.38 (Table S2). This suggests that to maintain sensitivity for *HER2* amplification in samples with a higher contamination with normal cells/DNA, a lower ratio than 2.0 could be utilized. A ratio of 1.5 would maintain the same degree of specificity, whilst potentially allowing for normal DNA contamination.

Many of the common, and rare, mutations of breast cancer have now all been defined [3], [17], We are entering an era of molecular characterization, based on the assessment of somatic mutations [18]. As such, extraction of DNA from tumour specimens will become routine, and this may allow digital PCR based assessments of *HER2* status to enter routine practice. In this manuscript we provide proof-of-principle that a digital PCR assay has sufficient diagnostic accuracy.

## Supporting Information

Figure S1 ***HER2:EFTUD2*** **copy number concordance in aCGH data.** Publically available microCGH data from 311 primary breast cancers, for the genomic region on chromosome 17q from 30 Mb–50 Mb (with whole chromosome data in Supplementary Figure 2). Displayed on the left are the profiles from 65 *HER2* amplified cancers and on the right 246 *HER2* non-amplified cancers. The genomic positions of *ERBB2* (*HER2*) and *EFTUD2* are marked. *HER2* amplification does not extend to *EFTUD2*, with *EFTUD2* stable in copy number with *HER2* in non-amplified cancers.
(TIF)

Figure S2 **Whole chromosome aCGH data for 311 primary breast cancers.** Publically available whole chromosome data from 65 *HER2* amplified cancers (left) and 246 *HER2* non-amplified cancers (right). The genomic positions of *ERBB2* (*HER2*), *EFTUD2* and the centromere are marked.
(TIF)

Table S1 DNA analysis of 11 tumours by two different ddPCR assays to check for reproducibility.
(XLS)

Table S2 ddPCR raw data obtained for all the samples employed in this study.
(XLS)
