# Supplementary figures and images for: Determination of HER2 Amplification Status on Tumour DNA by Digital PCR
Source: PLoS One. 2013 Dec 26;8(12):e83409. doi: 10.1371/journal.pone.0083409 (PMC3873285; doi:10.1371/journal.pone.0083409)

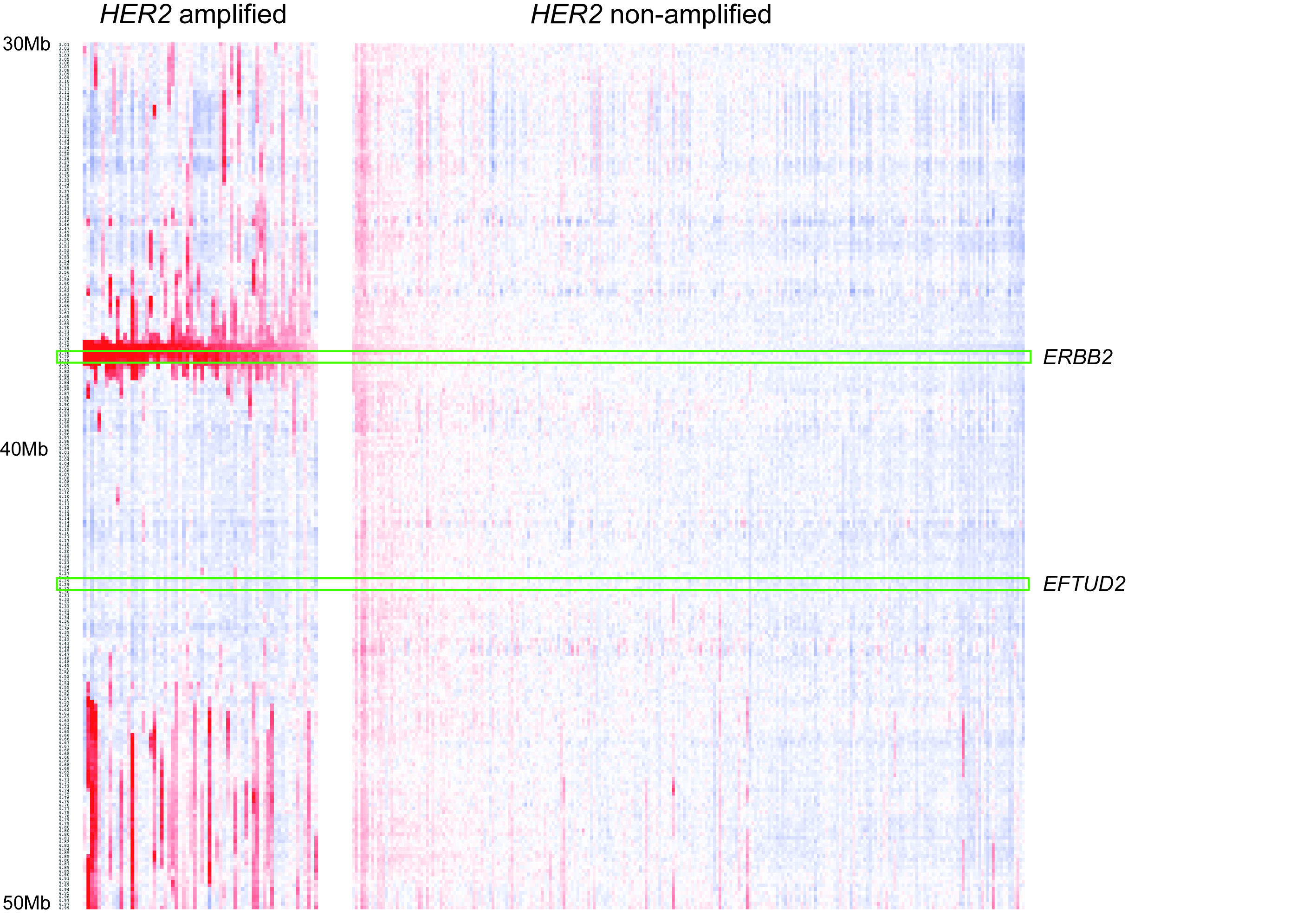

Supplement: Figure S1 — HER2:EFTUD2 copy number concordance in aCGH data. Publically available microCGH data from 311 primary breast cancers, for the genomic region on chromosome 17q from 30 Mb–50 Mb (with whole chromosome data in Supplementary Figure 2). Displayed on the left are the profiles from 65 HER2 amplified cancers and on the right 246 HER2 non-amplified cancers. The genomic positions of ERBB2 (HER2) and EFTUD2 are marked. HER2 amplification does not extend to EFTUD2, with EFTUD2 stable in copy number with HER2 in non-amplified cancers. (TIF) [file pone.0083409.s001.tif]

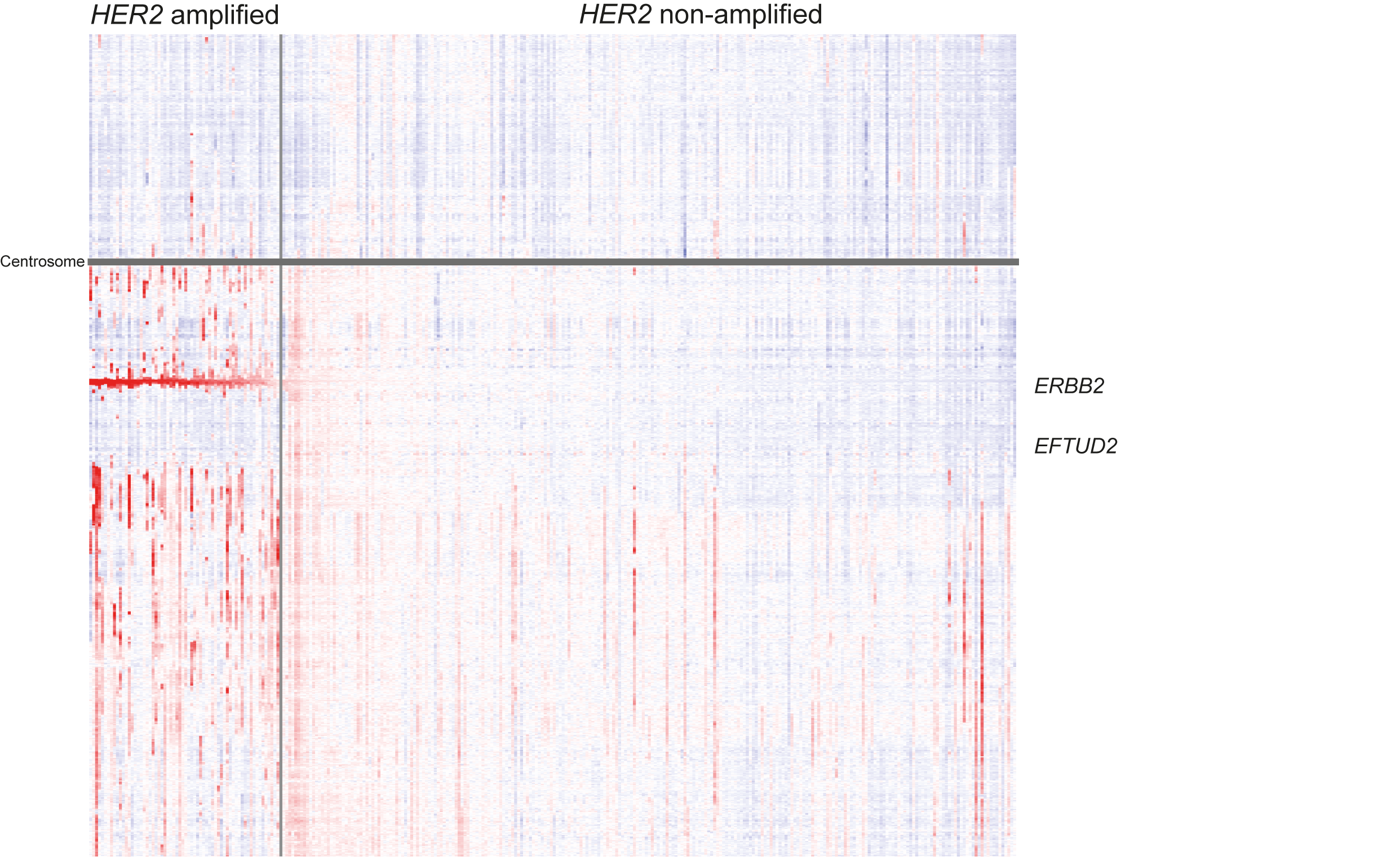

Supplement: Figure S2 — Whole chromosome aCGH data for 311 primary breast cancers. Publically available whole chromosome data from 65 HER2 amplified cancers (left) and 246 HER2 non-amplified cancers (right). The genomic positions of ERBB2 (HER2), EFTUD2 and the centromere are marked. (TIF) [file pone.0083409.s002.tif]
